# Nanoplasmonic NO_2_ Sensor with a Sub-10 Parts per Billion Limit of Detection in Urban Air

**DOI:** 10.1021/acssensors.1c02463

**Published:** 2022-03-31

**Authors:** Irem Tanyeli, Iwan Darmadi, Martin Sech, Christopher Tiburski, Joachim Fritzsche, Olof Andersson, Christoph Langhammer

**Affiliations:** † Department of Physics, Chalmers University of Technology, 412 96 Göteborg, Sweden; ‡ Insplorion AB, Arvid Wallgrens Backe 20, 413 46 Göteborg, Sweden

**Keywords:** nanoplasmonic sensor, WO_3_, NO_2_, air quality, parts per billion, urban air

## Abstract

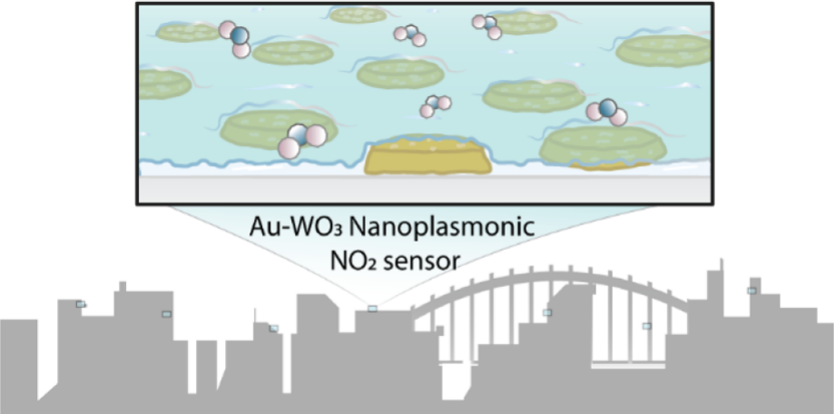

Urban air pollution is a critical health problem in cities all around the world. Therefore, spatially highly resolved real-time monitoring of airborne pollutants, in general, and of nitrogen dioxide, NO_2_, in particular, is of utmost importance. However, highly accurate but fixed and bulky measurement stations or satellites are used for this purpose to date. This defines a need for miniaturized NO_2_ sensor solutions with detection limits in the low parts per billion range to finally enable indicative air quality monitoring at low cost that facilitates detection of highly local emission peaks and enables the implementation of direct local actions like traffic control, to immediately reduce local emissions. To address this challenge, we present a nanoplasmonic NO_2_ sensor based on arrays of Au nanoparticles coated with a thin layer of polycrystalline WO_3_, which displays a spectral redshift in the localized surface plasmon resonance in response to NO_2_. Sensor performance is characterized under (i) idealized laboratory conditions, (ii) conditions simulating humid urban air, and (iii) an outdoor field test in a miniaturized device benchmarked against a commercial NO_2_ sensor approved according to European and American standards. The limit of detection of the plasmonic solution is below 10 ppb in all conditions. The observed plasmonic response is attributed to a combination of charge transfer between the WO_3_ layer and the plasmonic Au nanoparticles, WO_3_ layer volume expansion, and changes in WO_3_ permittivity. The obtained results highlight the viability of nanoplasmonic gas sensors, in general, and their potential for practical application in indicative urban air monitoring, in particular.

Ensuring a healthy and livable urban environment is a priority all over the world due to rapidly progressing urbanization. According to the WHO, air pollution, in general, and nitrogen dioxide (NO_2_), in particular, are among the largest health risk factors.^1^ As a consequence, the real-time monitoring of airborne pollutants, such as NO_2_, is of utmost importance to reliably assess their impact, to enable crafting and accurate evaluation of new policies, and for decision makers to take fast action in response to local air pollution episodes, such as real-time traffic congestion control. To monitor air quality, to date, highly accurate but costly, stationary and bulky measurement stations are used,^2^ and chemiluminescence has been defined as the standard NO_2_ measurement method in the corresponding European Standard (EN 14211: 2012). The data gathered by such monitoring stations provide high accuracy but offers only very low spatial resolution since these stations are very sparsely deployed at a few locations only due to their high cost. Hence, deeper insights into highly resolved spatial and temporal variability of pollutants remain impossible. Consequently, a technological breakthrough enabling—ideally—equally accurate but mobile and spatially highly resolved air quality monitoring devices are needed. To this end, one of the remaining key challenges is the required detection limit for NO_2_ in the low parts-per-billion (ppb) range^1^ and in the presence of potentially interfering molecular species abundant in urban air, such as O_2_, CO_2_, CO, and H_2_O. Therefore, significant research has been invested in developing NO_2_-sensing platforms comprising different materials and utilizing different readout principles, as summarized in recent reviews.^3,4^ Among the NO_2_-sensitive materials, metal oxides, in general, and tungsten trioxide (WO_3_), in particular, have been identified as highly NO_2_-selective and have therefore been explored in a plethora of designs, ranging from thin films to colloidal nanoparticles.^5−10^ Among a large number of sensor readout principles, resistive metal-oxide-semiconductor (MOS-type) sensors^11,12^ and electrochemical sensors^4,13^ are to date considered the best compromise in terms of technology maturity, sensitivity, cost, and device miniaturization potential. However, the performance of MOS-type sensors is limited by their long response time and signal drift, whereas electrochemical sensors are limited by cross-sensitivity and susceptibility toward changes in the humidity level and temperature.^14^ At the same time, nanoplasmonic gas sensors based on localized surface plasmon resonance (LSPR)^15,16^ have recently emerged as a competitive technology platform with high sensitivity, fast response, and significant miniaturization potential, in principle, down to the level of the individual nanoparticle.^17−19^ In the context of NO_2_ sensing, a proof-of-principle plasmonic detection combined with an NO_2_-selective material, such as a metal oxide^20−23^ or a molecular compound,^18,24^ has been demonstrated. However, no reports about the application of plasmonic NO_2_ sensors in real urban air exist, and their limit of detection (LoD) is generally widely unexplored.

Here we report a nanoplasmonic NO_2_ sensor platform based on arrays of Au nanoparticles coated with a thin layer of highly polycrystalline WO_3,_ for which we assess in detail its response to NO_2_ under (i) idealized laboratory conditions, (ii) conditions simulating humid urban air and (iii) in a realistic field test in the city of Göteborg, Sweden, benchmarked with a stationary chemiluminescence-based nitrogen oxide analyzer (Serinus 40, Acoem). As the key results, we find an extrapolated sensor LoD of about 3 ppb in all conditions, including the field test. This performance exceeds^5,25−28^ or is on par^9,29−33^ with the most sensitive NO_2_ sensors reported in the literature. Furthermore, together with the highly promising field test results, our findings highlight the potential of nanoplasmonic air quality sensors for large-scale deployment in urban environments for the purpose of so-called indicative monitoring of urban air.^34^ Such indicative monitoring serves the purpose of identifying the periods and spatial distribution of elevated NO_2_ concentrations with high spatial resolution and is, therefore, to be seen as a complement to, rather than a replacement of, the highly accurate measurement stations used to date.

## Results and Discussion

### Sensor Nanofabrication and Characterization

**Figure 1:**
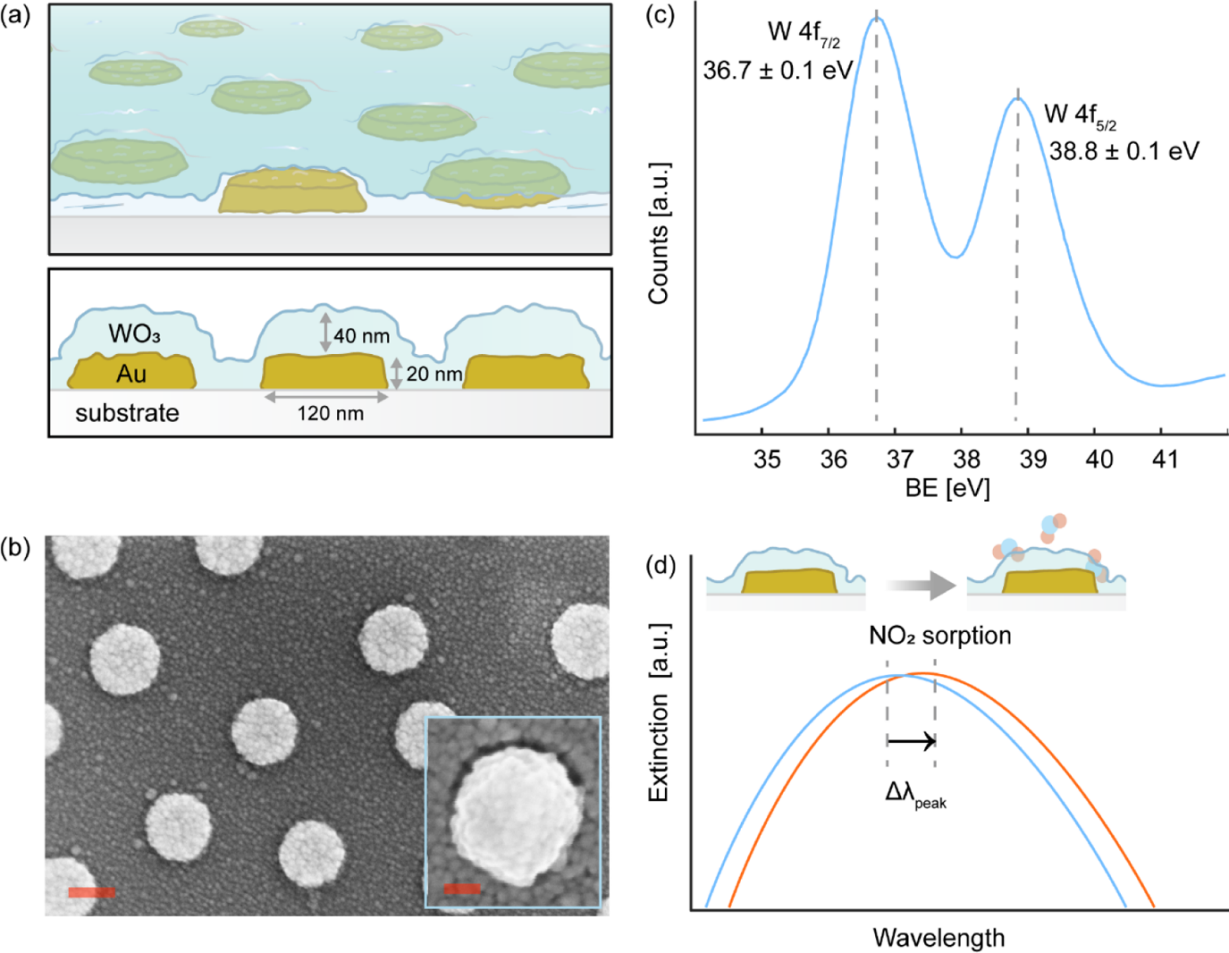
(a) Schematic top view and cross-section through the Au–WO_3_ nanoplasmonic sensor depicting the quasirandom array of Au nanodisks fabricated onto a transparent Borofloat glass substrate and encapsulated by a 40 nm thick WO_3_ film. (b) SEM image of a sensor surface revealing the WO_3_-coated Au nanodisks and the highly polycrystalline WO_3_ coating. Scale bar equals 100 nm. Inset: Zoom-in SEM image of a single WO_3_-coated Au nanodisk, scale bar equals 20 nm. (c) High-resolution XPS spectrum of the annealed sensor surface in the energy region of the W 4f_7/2_ and W4f_5/2_ doublet peaks, whose maxima are positioned at 36.7 ± 0.1, 38.8 ± 0.1 eV, respectively, which is in good agreement with a WO_3_ surface oxidation state.^36^ (d) Optical extinction spectra of a nanoplasmonic Au–WO_3_ sensor before (blue) and after (red) exposure to 1 part per million (ppm) NO_2_ in dry synthetic air. The interaction with NO_2_ induces a spectral redshift, Δλ_peak_, of the LSPR peak.

The sensor surfaces were prepared by nanofabricating a quasirandom array of Au nanodisks 120 nm in diameter and 20 nm in thickness onto a 9.5 × 9.5 × 1 mm glass support (Borofloat, Schott Scandinavia AB) using Hole-mask Colloidal Lithography^35^ (details in Methods). To functionalize it for NO_2_ detection with high specificity, we deposited a 40 nm thick WO_3_ film onto the nanostructured surface by RF magnetron sputtering, followed by two-step annealing at 400 °C for 12 h in 4% H_2_ in Ar, and subsequently at 400 °C for 12 h in air. This resulted in Au nanoparticles completely encapsulated in a highly polycrystalline layer (Figure 1a,b), for which X-ray photoelectron spectroscopy (XPS) analysis reveals that the W 4f_7/2_ and W 4f_5/2_ doublet peaks are positioned at 36.7 ± 0.1, 38.8 ± 0.1 eV, respectively. This confirms an oxidation state of the surface that corresponds to WO_3_ (Figure 1c).^36^ Exposing this sensor surface to NO_2_ then indeed results in a spectral shift of the LSPR peak, Δλ_peak_, which can be employed as the basis for the sensor readout to detect NO_2_ (Figure 1d).

### NO_2_-Sensing Mechanism

When it comes to using WO_3_ for the detection of NO_2_ in oxygen-rich environments, such as ambient air, the corresponding sensing mechanism has been reported in the literature based on both experimental and theoretical investigations and for different signal-transducing principles.^37−40^ These principles all have in common that they exploit the fact that oxygen molecules strongly interact with metal oxide surfaces, in general, and with WO_3_, in particular, according to the following scheme
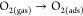
1
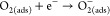
2
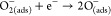
3

**Figure 2:**
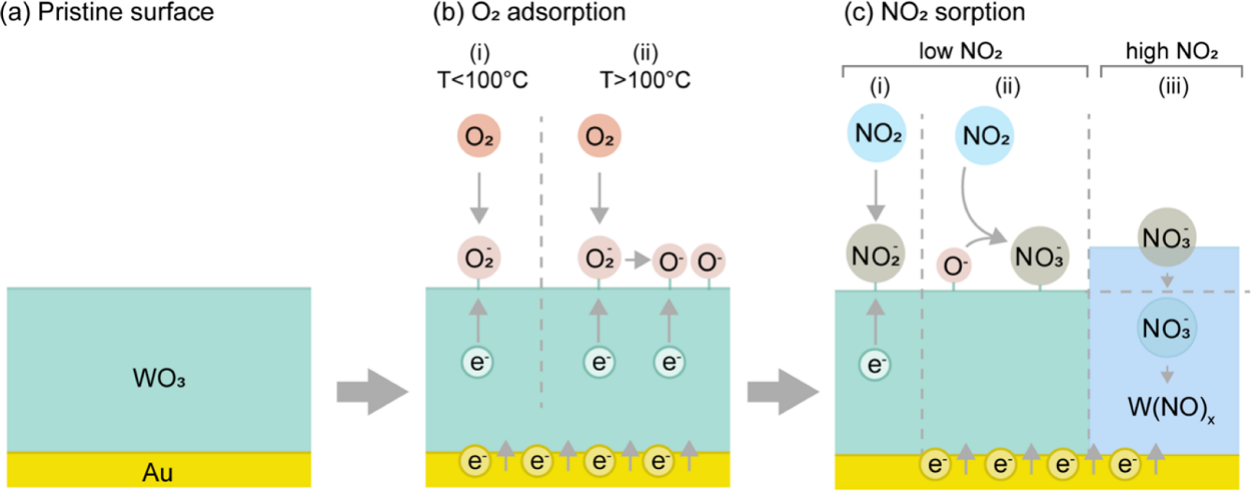
Schematic depiction of the proposed NO_2_-detection mechanism of Au–WO_3_ nanoplasmonic sensors. (a) Schematic of the pristine WO_3_ film on the top of the plasmonic Au nanodisk. (b) Schematic of oxygen adsorption on the Au–WO_3_ surface below and above 100 °C. At *T* < 100 °C, oxygen is adsorbed as O_2_^–^ by withdrawing an electron (e^–^) from the WO_3_, whereas at *T* > 100 °C, the adsorbed O_2_^–^ withdraws e^–^ and dissociates into 2O^–^. (c) Schematic of NO_2_ (ad)sorption in the ppb (low) and ppm (high) NO_2_ concentration regimes. NO_2_ is chemisorbed as NO_2_^–^ (nitrite) and NO_3_^–^ (nitrate) species by withdrawing electrons from the oxide and/or coadsorbed oxygen species, thereby changing the electron density in the oxide. This process, in turn, induces a charge equilibration between the oxide and the Au nanoparticles embedded in it, which lowers the electron density in the Au and gives rise to the observed spectral redshift of the LSPR peak. Furthermore, in the ppm (high) NO_2_ concentration range, besides charge transfer induced by the surface reaction, likely changes in the bulk of the metal oxide also have to be considered. Specifically, as a consequence of higher equilibrium NO_*x*_ surface coverage, a subsurface transformation of WO_*x*_ into W(NO)_*x*_ is likely to take place and leads to both a volume expansion and permittivity change of the oxide.

Here, depending on the operating temperature, different oxygen species are predominantly adsorbed on a WO_3_ surface, that is, for temperatures below 100 °C, it is mostly O_2_^–^ that captures electrons from the WO_3_ conduction band, and in the range from 100 to 300 °C, oxygen is mainly adsorbed in the form of O^–^ (Figure 2a,b).^41^

Introducing also NO_2_ to the system leads to the coadsorption of O_2_ and NO_2_ ions. However, owing to the five times higher electron affinity of NO_2_ compared to O_2_,^42^ NO_2_ chemisorbs in the forms of NO_2_^–^ (nitrite ion) or NO_3_^–^ (nitrate ion) by capturing electrons either from WO_3_ or from preadsorbed oxygen species, according to the following reactions^43^
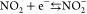
4
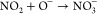
5

Since thereby an electron transfer from the surface to the analyte molecules takes place, the electrical conductivity of the active metal-oxide-sensing layer is altered, enabled by the existence of native vacancies and defects in its structure. The specific role of these defects in WO_3_-based NO_2_ detection has been investigated in detail in various studies.^39,40,44^ The common conclusion is that the interaction between WO_3_ and NO_2_ is enhanced in the presence of the oxygen vacancies since they function as active adsorption sites for NO_2_.^45^ Consequently, the majority of reported WO_3_-based NO_2_ sensors are of the MOS-type, in which measured changes in the conductivity of the WO_3_-sensing layer in the presence of NO_2_ constitute the signal transduction principle.^5,9,46^ Accordingly, also other oxides like ZnO,^47,48^ SnO_2_,^49,50^ and In_2_O_3_^51,52^ have been used in MOS-type NO_2_ sensors by exploiting a similar detection principle.

In this study, however, we utilize a different sensing principle, which on the one hand relies on the strong interaction of the Au nanodisk array on the sensor surface with incident visible-NIR light via LSPR, and on the other hand, the sensitivity of the LSPR to changes occurring both to the plasmonic nanoparticles themselves and to their intimate surroundings, which subsequently is reflected in a finite Δλ_peak_ (cf. Figure 1d). To specifically rationalize the origin of the observed Δλ_peak_ signal generated by NO_2_ for the sensor surface at hand, we recall that the LSPR frequency of a Au nanoparticle, Ω, in its simplest form, is a function of the free electron density in the metal and the refractive index of the surrounding matrix as
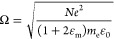
6where *N* is the conduction electron density, *e* is the elementary charge, ε_m_ is the dielectric function of the matrix, *m*_e_ is the electron mass, and ε_0_ is the permittivity of free space.^21,53^ Translated to the case at hand, the chemisorption of NO_2_ onto the WO_3_ surface leads to a conductivity change of the WO_3_ layer due to electron depletion by the formed NO_*x*_^–^ species on its surface, as discussed above. Consequently, owing to a subsequent charge equilibration between the WO_3_ layer and the Au nanoparticles, the free electron density of these particles is slightly reduced and leads to the observed spectral redshift of the LSPR peak, as also proposed in the literature for other Au—metal oxide nanocomposite plasmonic gas sensors.^21,54^

Next, it is also interesting to briefly consider the likely impact of NO_2_ concentration in the analyte medium on this process. For low NO_2_ concentrations in the ppb range, the equilibrium coverage of NO_*x*_^–^ is low and likely limited to the surface, rendering charge transfer from the Au nanodisks to surface-bound chemisorbed NO_*x*_^–^ via WO_3_, the main sensing mechanism (Figure 2c). However, when the NO_2_ concentration in the analyte medium increases to the parts per million (ppm) range, the equilibrium NO_2_^–^ and NO_3_^–^ coverage on the sensor surface increases significantly, and the formation of NO_3_^–^ is favored,^55^ as observed on various metal oxides in experimental studies and corroborated by theoretical calculations.^56,57^ Since adsorbed NO_3_^–^ species are also known to have a higher stability than adsorbed NO_2_^–^, it becomes increasingly likely that a subsurface transformation of WO_*x*_ into W(NO)_*x*_ also takes place at high NO_*x*_ concentrations in the analyte medium (Figure 2c). Since this process not only leads to a charge transfer but also induces a volume expansion and a sizable change in permittivity of the oxide matrix around the Au nanoparticles (both of unknown magnitude since no corresponding studies determining their magnitude exist to the best of our knowledge), the observed plasmonic response at higher NO_2_ concentrations is likely a cumulative effect of three factors, that is, (i) charge transfer, (ii) matrix volume expansion, and (iii) matrix permittivity change (Figure 2c).

In addition, we note that it is likely that the sputtered WO_3_ layer exhibits a certain degree of porosity. In principle, this means that such pores may enable NO_*x*_ diffusion to the Au/WO_3_ interface and thus direct interaction between Au and NO_*x*_ that may contribute to or even provide a complementary sensing mechanism. However, as our control experiments on uncoated Au nanoparticles reveal, even at high NO_2_ concentrations in the 5–10 ppm range, no significant Δλ_peak_ response is recorded (Figure S1), which corroborates the sensing mechanism discussed above.

### NO_2_ Detection in Dry Synthetic Air

**Figure 3:**
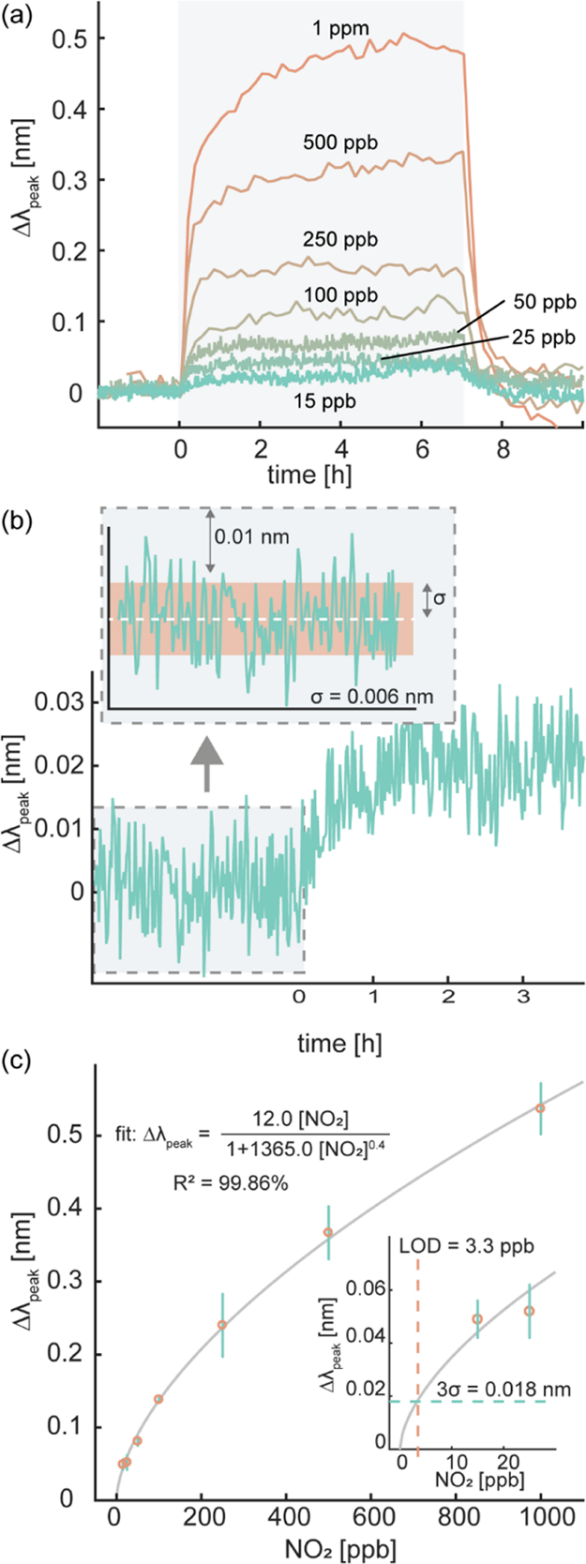
(a) Time-resolved Δλ_peak_ response of a Au–WO_3_ nanoplasmonic sensor to NO_2_ exposures at different concentrations in dry synthetic air at 250 °C. The shaded area denotes the pulse of NO_2_ exposure with the specific concentrations indicated in the figure. The different noise levels between high and low NO_2_ concentrations are due to different data acquisition sampling times. (b) Zoom-in on the Δλ_peak_ response of the sensor to 15 ppb NO_2_. Inset: noise level determination, revealing a standard deviation (σ) of 0.006 nm, as denoted by the red band. (c) Δλ_peak_ of the sensor plotted as a function of the NO_2_ concentration. The error bars denote the standard deviation from three exposure pulses at each NO_2_ concentration. The solid line depicts a fit to the experimental data using the Redlich–Peterson semiempirical adsorption model.^58^ The inset shows the same plot for the low end of the NO_2_ concentration range. The green- and red-dashed lines signify the three-fold noise level (3σ = 0.018 nm) and the extrapolated limit of detection (LOD = 3.3 ppb), respectively.

To test the sensing performance toward NO_2_ in dry laboratory conditions, we first conditioned an as-fabricated and thermally annealed sensor by exposing it for 4 h to synthetic air at 250 °C. After this conditioning stage, we conducted NO_2_-sensing measurements from the 1 ppm down to 15 ppb NO_2_ concentration range (the lowest concentration attainable with our setup) by exposing the sensor to different NO_2_ pulses with different concentrations in synthetic air at 250 °C (Figure 3a). Each concentration step was repeated 3–4 times (Figure S2). Evidently, the sensor exhibits a consistent, reversible, and reproducible response that distinctly depends on NO_2_ concentration. Furthermore, a typical noise level of σ = 0.006 nm can be extracted from the sensor response (Figure 3b). To determine the concentration dependence of this response and thereby generate a calibration curve, we extracted Δλ_peak_ for all measured NO_2_ pulses and plot them as a function of NO_2_ concentration (Figure 3c). This analysis reveals a distinct concentration dependence of Δλ_peak_ and an extrapolated LoD of ca. 3 ppb at these idealized dry conditions in synthetic air. It is also worth noting that the error bars at higher NO_2_ concentrations are larger than at lower concentrations. This is likely the consequence of our measurement sequence implemented from high to low NO_2_ concentration (Figure S2) since the sensor is “fresh” at the first high concentration exposures and, therefore, initially undergoes a certain degree of structural conditioning during the first exposures to NO_2_ before reaching a new morphological equilibrium state.

### Temperature Dependence of Sensor Response in Dry Synthetic Air

**Figure 4:**
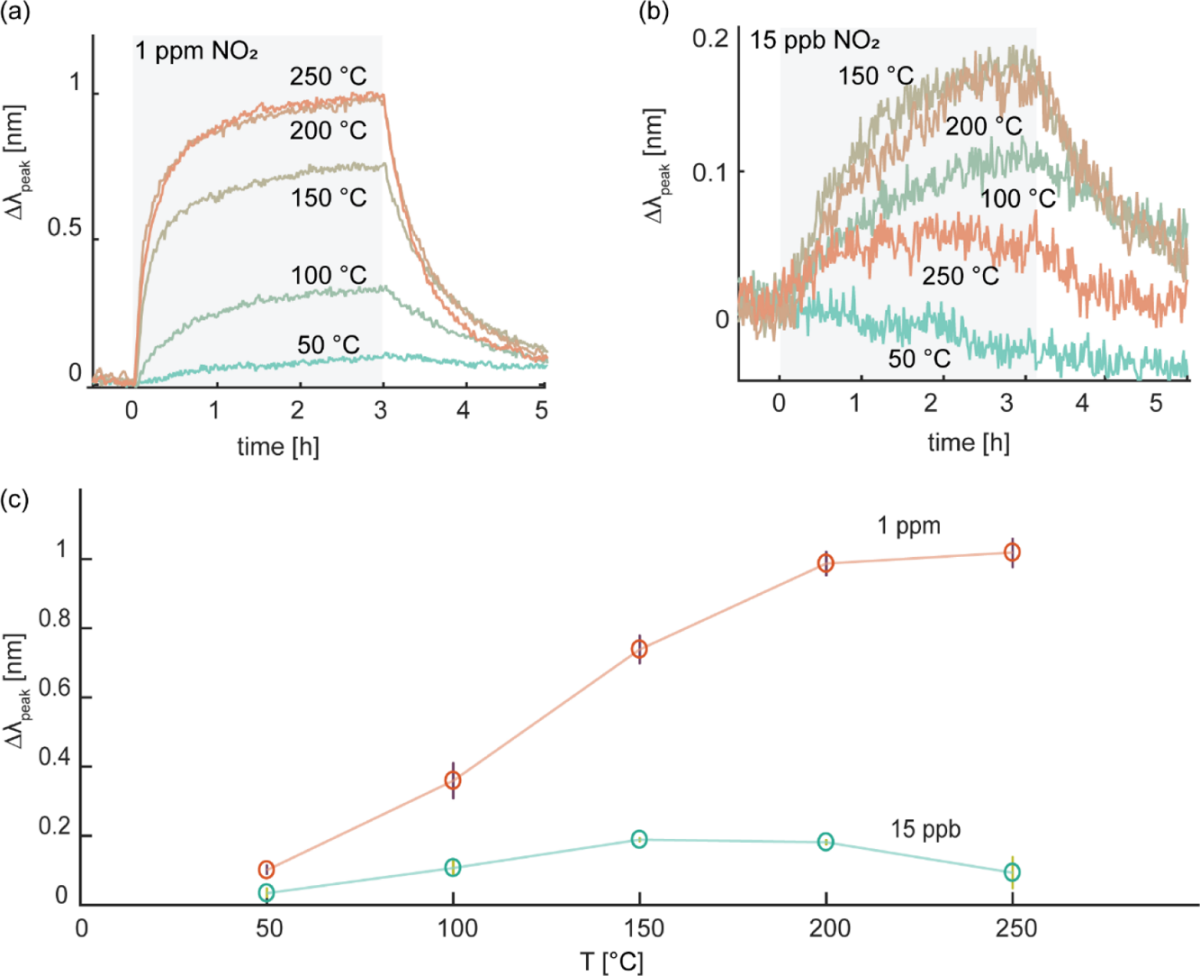
(a) Time-resolved Δλ_peak_ response toward 1 ppm NO_2_ plotted as a function of operating temperature in the range 50–250 °C. (b) Time-resolved Δλ_peak_ response toward 15 ppb NO_2_ plotted as a function of operating temperature in the range of 50–250 °C. The shaded areas depict the NO_2_ pulse. (c) Δλ_peak_*vs* operating temperature as obtained from (a,b). The error bars denote the standard deviation from three subsequent NO_2_ pulses at each temperature. We note that the different absolute Δλ_peak_ value at 250 °C compared to Figure 3a is a consequence of batch-to-batch variation since the sensor investigated here was made as part of a different batch than the one used to obtain the data displayed in Figure 3a.

The operating temperature has been reported to have a significant impact on the NO_2_-sensing performance of WO_3_.^41,46,59^ Hence, it is important to characterize our system in this respect. To do so, we investigated the sensors in dry synthetic air in the temperature range from 50 to 250 °C, with 50 °C increments, using both the highest and lowest NO_2_ concentrations of our measurement range, that is, 1 ppm and 15 ppb. Focusing first on the high concentration 1 ppm pulses, Δλ_peak_ increases significantly with temperature up to 200 °C. Then, we don’t observe a further Δλ_peak_ increase when ramping up the operating temperature to 250 °C (Figure 4a). Interestingly, a different trend is revealed for the 15 ppb case, for which we record no response at 50 °C and a maximum amplitude at 150 °C before decreasing again at even higher temperatures (Figure 4b).

To rationalize the identified significantly different temperature dependencies of the sensor at 15 ppb and 1 ppm (Figure 4c), we remind ourselves that both equilibrium surface coverages of chemisorbed species and reaction kinetics are temperature dependent. Generally, adsorption/desorption equilibria are shifted in favor of desorption at a higher temperature, which means that adsorbate surface coverages usually are lower at higher temperature.^60,61^ At the same time, reaction kinetics are enhanced at elevated temperatures, and more bulk-like W(NO)_*x*_ phases may form also in the subsurface region of the WO_3_ layer.^55,62^ Translated to our situation, this means that the former effect is expected to be most prominent in the low NO_2_ concentration regime, where the sensor response is expected to be solely dictated by NO_*x*_^–^ coverage on the surface, and thus explains why we observe a signal amplitude maximum at 150 °C (Figure 4b,c). At higher NO_2_ concentrations in the ppm range, on the other hand, the temperature dependence of the NO_*x*_^–^ surface coverage is expected to be significantly less pronounced as the surface is expected to be completely covered in the considered temperature range. Therefore, in this regime, reaction kinetics for the formation of W(NO)_*x*_ become more relevant and the dominating factor that dictates the sensor response amplitude, thereby explaining the observed continuous Δλ_peak_ increase for the increasing temperature at 1 ppm NO_2_, as well as the generally accelerated response (Figure 4a,c). As the main conclusion, we thus identify a sensor operation temperature of 150 °C as the best compromise for a wide dynamic range and use it from here forward.

### NO_2_ Detection in Simulated Humid Urban Air

**Figure 5:**
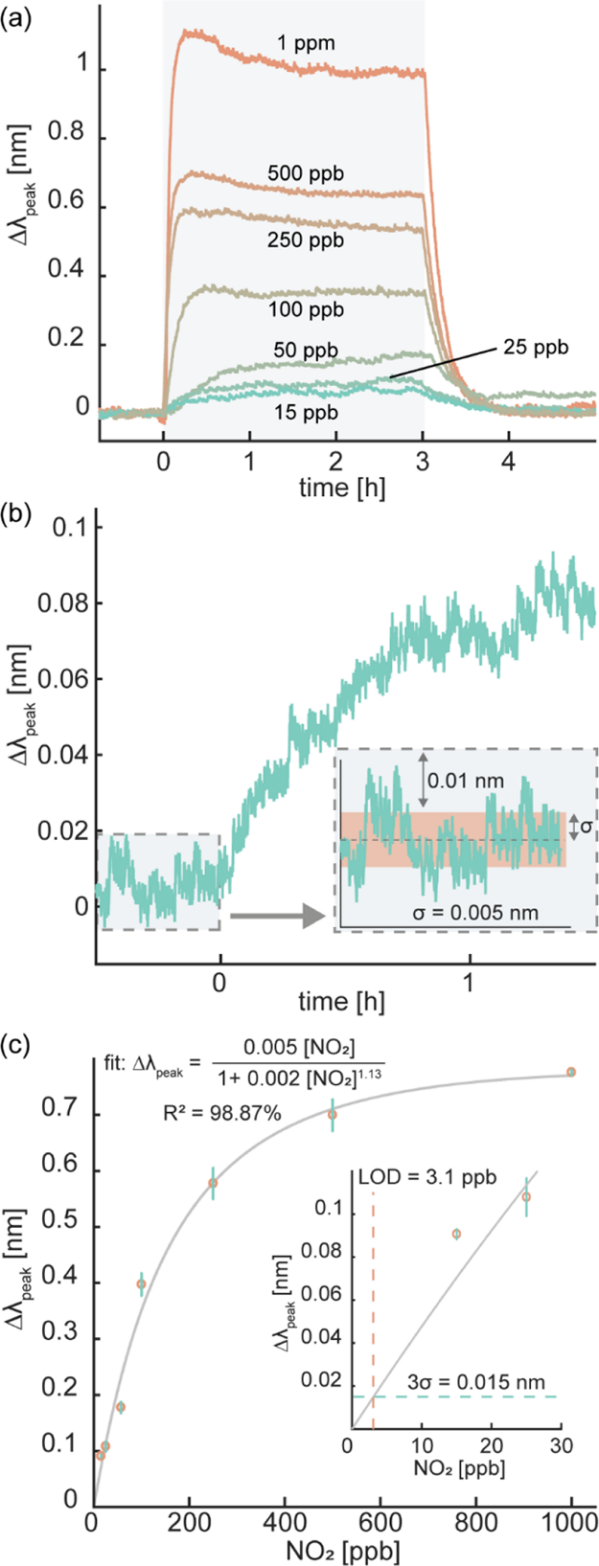
(a) Time-resolved Δλ_peak_ response of a nanoplasmonic Au–WO_3_ sensor upon exposure to different NO_2_ concentration pulses in synthetic air mixed with 1 ppm CO, 400 ppm CO_2_, and 50% RH set at 30 °C. The sensor operating temperature was 150 °C. The shaded area denotes the NO_2_ pulse duration. (b) Zoom-in on the time-resolved Δλ_peak_ response of the sensor to 15 ppb NO_2_ from (a). Inset: noise level determination revealing a standard deviation (σ) of 0.005 nm, as denoted by the red band. (c) Δλ_peak_ of the sensor plotted as a function of NO_2_ concentration. The error bars denote the standard deviation from three pulses at each NO_2_ concentration. The solid line depicts a fit to the experimental data using the Redlich–Peterson semiempirical adsorption model.^58^ The inset shows the same plot for the low end of the NO_2_ concentration range. The green- and red-dashed lines signify the threefold noise level (3σ = 0.015 nm) and the extrapolated limit of detection (LoD = 3.1 ppb), respectively.

To further benchmark our nanoplasmonic Au–WO_3_ sensor platform for air quality monitoring in urban air, we designed an experiment that closely resembles real ambient conditions. Specifically, we operated the system in synthetic air mixed with 1 ppm CO and 400 ppm CO_2_, humidified to 50% relative humidity (RH) at 30 °C, to emulate urban air at ambient conditions, where the CO and CO_2_ concentrations mimic the natural abundance of these species. Like in the previous experiments, we then exposed the sensor to NO_2_ pulses at concentrations ranging from 1 ppm down to 15 ppb (Figure S3), with the sensor heated to 150 °C that we identified above as the best compromise in terms of sensitivity toward both high and low NO_2_ concentrations (Figure 5a). As the main result, we observe a distinct, reversible, and NO_2_ concentration-dependent Δλ_peak_ response down to 15 ppb, which again is the lowest concentration we can produce in our setup. This is a remarkable performance since it is achieved despite potential cross-sensitivity to the background species in the gas mixture.^63−65^

To this end, while a detailed assessment of the role of these different molecular species in the sensing process is beyond the scope of our study, an earlier study has revealed complex surface chemistry as a consequence of the fact that CO_2_, H_2_O, and NO_2_ are oxidizing, whereas CO is a reducing gas. This, for example, means that they either may compete for or assist with the adsorption of NO_2_ on the surface.^66^ As the key point here, however, we clearly find that the presence of these molecules does not impair sensor performance in terms of the magnitude of the Δλ_peak_ response since 15 ppb NO_2_ is easily resolved, just like in the dry case, without CO and CO_2_ (Figure 5a). In fact, by determining the typical noise in our sensor response as σ = 0.005 nm (Figure 5b) and then extrapolating the Δλ_peak_*versus* NO_2_ concentration curve in the low concentration range, we can derive an LoD defined by three times the typical noise, 3σ, of ca. 3.1 ppb, which is identical to a sensor operated at dry conditions and without CO and CO_2_ in the background (Figure 5c).

To put this result into perspective, we first note that an LoD of 3 ppb is on par with the best thin-film WO_3_-based MOS-type NO_2_ sensors reported in the literature.^9,33^ However, as the key distinctive feature and a step beyond this state of the art, our sensors exhibit this low ppb LoD in an environment where all molecular species are mixed (and not where the sensor is exposed sequentially to them^9,33^), thereby truly emulating a real urban air environment.

### Field Testing a Nanoplasmonic Au–WO_3_ NO_2_ Sensor

As the last step of our Au–WO_3_ nanoplasmonic sensor chip benchmarking, we integrated it with a miniature urban air quality sensor device (Insplorion AB, Göteborg, Sweden) to test its NO_2_-detection performance in real urban air in a proper field test. To generate the sensor readout, the device measures the relative change in transmitted light intensity by the sensor chip over a range of wavelengths in the red/NIR spectral region. The specific wavelength range is chosen to coincide with the left flank of the LSPR peak of the sensors to maximize the transmittance change upon a shift of the peak^67^ induced by a change in NO_2_ concentration. To measure this transmittance change, standard light-emitting diodes and surface-mounted photodetectors are used in the device, and a microcontroller maintains the working temperature of the sensor chip constant at above 100 °C. The fractional increase in light transmitted through the sensor chip, caused by a redshift of the LSPR peak, is used as the signal readout.

**Figure 6:**
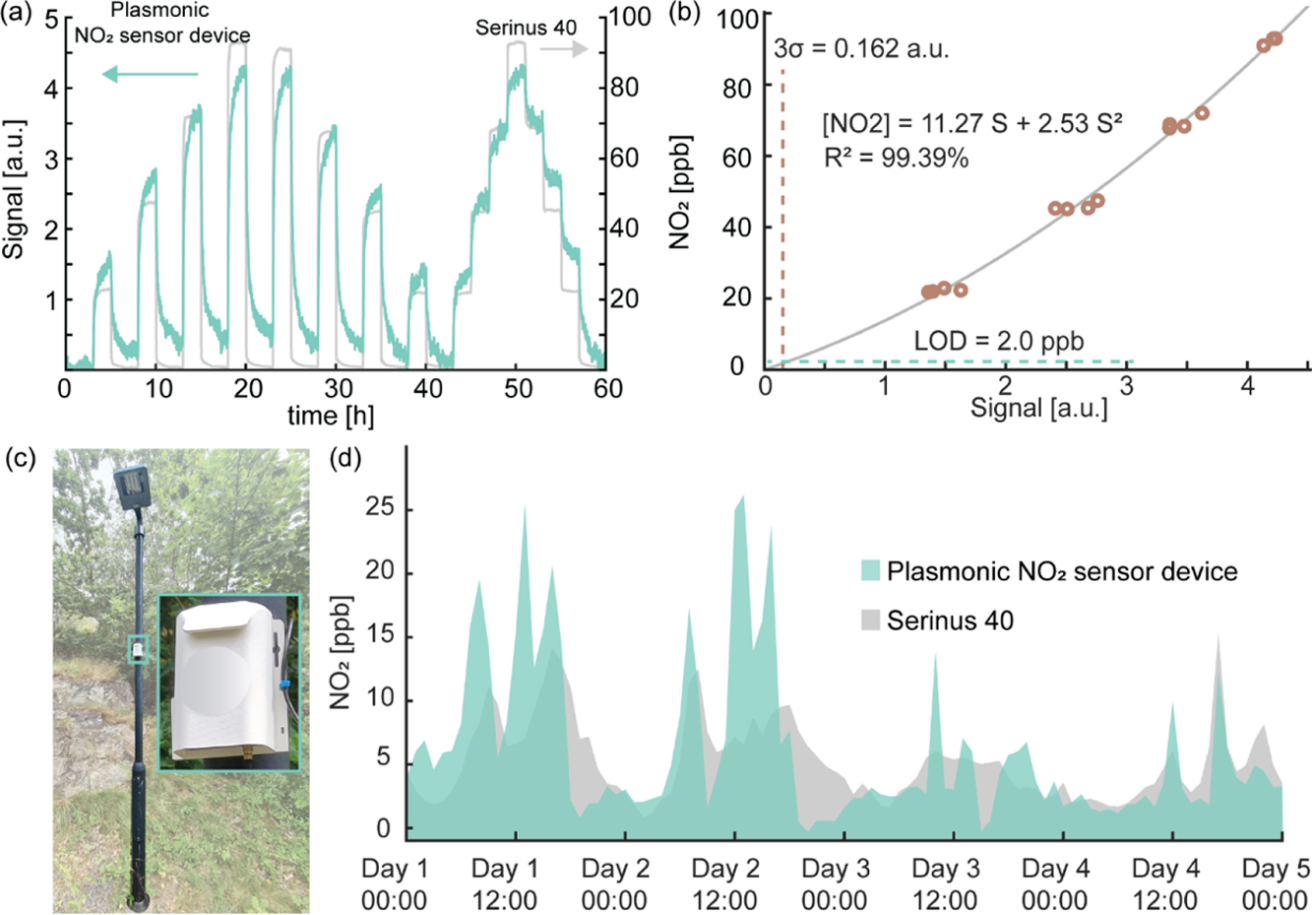
(a) Time-resolved Au–WO_3_ nanoplasmonic sensor device response to NO_2_ pulses and steps in the concentration range of 25–100 ppb benchmarked by a Serinus 40 chemiluminescence measurement system. (b) Corresponding Au–WO_3_ nanoplasmonic sensor calibration curve derived from the data shown in (a). It is used to derive the transfer function that converts the sensor response of the device into absolute NO_2_ concentration values by fitting a second-degree polynomial (with the intercept term set to zero) to the experimental data points. The extrapolated LoD is depicted by the dashed lines, and with 3σ = 0.162 a.u., it equals ∼2.0 ppb. (c) Photograph of the Au–WO_3_ nanoplasmonic sensor device mounted on a light pole for field testing close to a highly trafficked road in Göteborg, Sweden. (d) Direct comparison of the NO_2_ concentration evolution measured across the 5 day field test by the Au–WO_3_ nanoplasmonic sensor device (green) and the Serinus 40 reference system (grey). All data have been averaged to 15 min increments.

To calibrate the device prior to the field test measurements, we exposed it to multiple pulses and steps of NO_2_ in dry synthetic air in the concentration range of 25–100 ppb in the laboratory (Figure 6a). The obtained response plotted as a calibration curve is shown in Figure 6b. It indicates an extrapolated LoD of 2.0 ppb, which is on par with the LoD’s identified above for the sensor chips alone and using Δλ_peak_ as the readout (cf. Figures 3c and 5c). Based on this calibration curve, a transfer function relating the change in relative transmittance measured by the device and NO_2_ concentration was determined. The microcontroller in the device was then configured to automatically perform the transfer function during the field measurements to determine the NO_2_ concentration in real time.

The field test itself was conducted by sampling air from an urban environment in Göteborg, Sweden, over the span of 5 days by mounting the device close to a road with high traffic activity in the city (Figure 6c). As the main result, we obtained reliable real-time NO_2_ concentration measurements by the plasmonic NO_2_ sensor in a concentration range of ∼2–25 ppb, with a general rise of the ambient NO_2_ levels during daytime and with distinct peaks due to increased traffic activity (Figure 6d—all data have been averaged to 15 min increments). Remarkably, the measured general trends and absolute concentration values are in quite good agreement with the reference measurements executed simultaneously using the Serinus 40 reference station.

At the same time, we observe some discrepancies in the quantification of NO_2_ concentrations for some measurement periods. To put these into perspective, we first note that the Serinus 40 is a certified reference instrument that detects NO_2_ by chemiluminescence with high accuracy, whereas our sensor device has been developed with the intention to be used for indicative monitoring. In this sector, to date, no standards exist, and lower accuracy can be tolerated as a trade-off for the possibility to deploy miniaturized and cost-effective sensors with high spatial density across, for example, a city.

Nevertheless, despite this difference in scope of the two systems, it is important to discuss the potential reasons for the observed discrepancies. As the contributing first reason, we identify the different gas intake characteristics of the two systems. In the plasmonic system, the sensor surface is separated from the ambient air by a polytetrafluoroethylene membrane with 100 nm pore size, which means gas transport to the sensor surface is entirely reliant on diffusion, with the membrane being the bottleneck. The Serinus 40, in contrast, uses pneumatic ports for air sampling, which very likely creates very different mass transport characteristics in the two systems and, therefore, affects response and recovery times on the shorter time scales. These effects are, however, not severe enough to explain the major observed discrepancies between the two systems.

A second potentially important factor to consider is varying humidity during the field test due to weather variations in the course of the 5 day period. Here, in the Serinus 40, water is removed from the sampled air by Nafion tubing inside its dryer compartment, and the instrument, thus, always samples dry air, whereas in the plasmonic device, the ambient air is sampled as is. Hence, even though relative humidity changes occurring at ambient conditions due to weather variations are reasonably small when translated to the plasmonic sensor’s high operating temperature, they are likely still relevant. This hypothesis is corroborated by our laboratory measurements in humid synthetic air, which revealed faster response with larger amplitude per unit NO_2_ in humid (cf. Figure 5a) compared to dry (cf. Figure 3a) conditions. This, thus, suggests that (a part of) the discrepancy between the two sensor systems used in the field test may be the consequence of humidity variations.

As a final aspect, we note that in urban air, not only NO_2_ but also NO is present, however, usually at even lower concentrations. Therefore, it is relevant to briefly address the potential cross-sensitivity of our plasmonic sensor toward NO. Here, we can resort to that Serinus 40 also measured the NO concentration during the field test, yielding an average of a few ppb consistently below the NO_2_ level (Figure S4). Furthermore, a control experiment, where we exposed the plasmonic sensor to 2 and 3 ppm NO, revealed an opposite response, that is, a spectral blueshift of the LSPR peak (compared to a redshift for NO_2_) at essentially one order of magnitude smaller amplitude compared to the corresponding response to NO_2_ (Figure S5). This finding is in-line with similar studies performed with resistive metal oxide sensors^39,68^ and implies that variations in the NO concentration are likely negligible in the plasmonic sensor response in the NO concentration range identified for the field test and thus for air quality monitoring in general.

## Conclusions

In conclusion, we have presented a Au–WO_3_ nanoplasmonic NO_2_ sensor with a sub-10 ppb limit of detection both in laboratory conditions and in a 5 day field test next to a highly trafficked road in Göteborg, Sweden, using a miniaturized autonomous sensor device, which we also benchmarked with a chemiluminescence-based Serinus 40 reference system certified both according to European (EN14211) and US EPA (RFNA-0809-186) standards. The found performance of the Au–WO_3_ nanoplasmonic NO_2_ sensor, which is enabled by a nanofabricated sensor chip surface comprising a quasirandom array of Au nanodisks coated with a 40 nm thick polycrystalline WO_3_ film operated above 100 °C, is on par with or exceeds the performance of existing solutions using alternative readout principles in terms of the limit of detection. The identified discrepancies between the plasmonic sensor and the reference system during the field test are identified as likely consequences of humidity variations handled differently by the two systems and highlight the importance of further investigations of humidity-related effects. Taken all together, these results prove the viability of nanoplasmonic gas sensors, in general, and their potential for practical application in indicative urban air monitoring, in particular, where low cost and large-scale deployment capability are the key enabling factors.

## Methods

### Sensor Nanofabrication

Au nanodisk arrays were fabricated using the Hole-Mask Colloidal Lithography technique, which is described in detail elsewhere,^35^ onto 9.5 × 9.5 mm^2^ glass substrates (Borofloat, Schott Scandinavia) and silicon wafer substrates (for SEM imaging and XPS measurements). In brief, the hole-mask nanofabrication steps were as follows:

(1) Substrates were cleaned in an ultrasonic bath consecutively with acetone, isopropanol, and deionized water. Each step was applied for 3 min.
(2) A PMMA (MicroChem, 950 000 molecular weight, 2 wt % in anisole) layer was spin-coated at a spin rate of 2000 rpm for 45 s. Subsequently, the substrate was placed on a hot plate at 170 °C for 5 min for soft-baking.
(3) To reduce the hydrophobicity of the surface before drop-coating a suspension of positively charged poly(diallyldimethylammonium chloride) (PDDA) solution, the substrates were exposed to oxygen plasma (Plasma-Therm Batchtop RIE 95 m, 50 W, 250 mTorr, 10 sccm) for 5 s.
(4) A PDDA solution (Sigma-Aldrich, *M*_w_ = 200,0000–350,000, 0.2 wt % in Milli-Q water) was drop-cast onto the PMMA layer and incubated for 45 s, followed by rinsing in deionized water and blow-drying with nitrogen gas.
(5) A suspension of negatively charged polystyrene (PS) spheres (Interfacial Dynamics Corporation, 120 nm in diameter, 0.2 wt % in Milli-Q water) was drop-cast and incubated for 3 min, followed by rinsing in deionized water and blow-drying with nitrogen gas.
(6) A 15 nm thick chromium film was deposited by e-beam physical vapor deposition (Lesker PVD 225, base pressure of 5 × 10^–7^ Torr, deposition rate of 1 Å/s).
(7) The PS spheres were removed from the surface by tape-stripping (SWT-10 tape, Nitto Scandinavia AB) to reveal holes in the Cr film at the positions of spheres.
(8) To complete the hole-mask pattern, the surface was exposed to oxygen plasma (Plasma-Therm Batchtop RIE 95 m, 50 W, 250 mTorr, 10 sccm) for 3 min to etch the PMMA layer through the holes in the Cr film.
(9) A 20 nm thick gold film was deposited with the same technique and parameters used in step (6) to grow the Au nanodisks through the hole-mask.
(10) The samples were soaked in acetone to dissolve the remaining PMMA layer, rinsed in isopropanol, and blow-dried in nitrogen gas. This final step left the surface covered with gold nanodisks.
(11) A 40 nm thick WO_3_ thin film was RF-magnetron-sputtered onto the Au nanodisks using a power of 150 W and 1:1 Ar:O_2_ (30 sccm) at 25 mTorr.
(12) Two steps of annealing were applied as post-processing. The samples were annealed first at 400 °C for 12 h under the flow of 4% H_2_ in Ar in a tube furnace, followed by 400 °C for 12 h in air.

### Material Characterization

A Zeiss Supra 55 VP SEM was used for imaging sensor surfaces at an electron beam acceleration voltage of 10 kV using a secondary electron detector. For further material characterization, XPS measurements were executed in a PerkinElmer PHI 5000C ESCA system with an energy step width of 0.125 eV and a pass energy of 58.70 eV. The correction of peaks was done with respect to the carbon 1s peak using the Multipak 6.0 software.

### NO_2_-Sensing Measurements

The measurements were conducted in a quartz tube plug-flow reactor equipped with an optics unit for transmittance measurements (Insplorion X1, Insplorion AB). The resistive heating coils around the tube and Eurotherm temperature controller enable measurements at up to 600 °C. The standard deviation of the sensor temperature reading is ∼0.1 °C. The reactor was configured with several mass flow controllers (Bronkhorst Δ*P*) to regulate the gas compositions and with a humidifier (Bronkhorst-controlled evaporator and mixer) to mimic humid air. Synthetic air (Strandmöllen AB, 20.9% O_2_, 79.1% N_2_) was used as the carrier gas, and all the gases involved in the measurements (NO_2_, CO, CO_2_—Strandmöllen AB) were supplied from cylinders diluted in synthetic air. The total gas flow rate used in the experiments was 340 mL/min.

The sensor chip mounted in the reactor was illuminated by a tungsten halogen lamp (AvaLight-Hal, Avantes) through an optical fiber with a collimating lens, and the transmitted light was collected using a fixed grating spectrophotometer (AvaSpec-ULS2048CL-EVO, Avantes). A 20th degree polynomial fit is applied to the raw measured extinction spectra around the LSPR peak. The λ_peak_ is determined by finding the wavelength where the first derivative of the fitted polynomial is equal to zero. The shift in the λ_peak_ was used as the sensing descriptor in this study.

### Plasmonic NO_2_ Sensor Device Measurements in Laboratory Settings

The sensor device was exposed to pulses and steps of NO_2_ in dry synthetic air in the concentration range of 25–100 ppb, regulated by several mass flow controllers (Bronkhorst Δ*P*). Simultaneously, the NO_2_ concentration throughout the measurement was monitored by a stationary nitrogen oxide analyzer (Serinus 40, Acoem) using chemiluminescence technology. A calibration curve was derived by plotting the device signal for the corresponding NO_2_ concentration detected by the nitrogen oxide analyzer.

### Plasmonic NO_2_ Sensor Device Field Test Measurements

The sensor device was calibrated in laboratory settings prior to the field test measurements. The device was placed in a protective casing and mounted close to a road with high traffic activity in Göteborg, Sweden. The field test measurement was conducted over the span of 5 days. In order to compare the performance of the sensor device, the Serinus 40 nitrogen oxide analyzer was used to monitor the air in the vicinity of the device. NO_2_ concentrations measured by the device and the analyzer were averaged to 15 min increments.

The Serinus 40 reference instrument uses the gas-phase chemiluminescence technique to detect NO and NO_2_.^69^ The sample gas passes via two different paths— NO path and NO_*x*_ path. The NO_*x*_ path has a longer residence time due to a delay loop and an NO_2_ to NO converter. Any NO species passing through this path remains unaffected, whereas the NO_2_ species are converted into NO. Hence, the total amount of NO reaching the reaction cell is the combination of original NO present in the sample and converted NO_2_.

At the end of each path, the sample gas arrives at the reaction cell and reacts with ozone to form activated NO_2_ species (chemiluminescence reaction for NO).
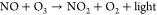
7

The luminescence of the activated NO_2_ species is detected by a photomultiplier tube. The NO concentration is evaluated from the intensity of the chemiluminescence. The NO_2_ concentration is calculated by subtracting the NO concentration obtained in the NO path from the NO_*x*_ path.

The instrument holds both US EPA (RFNA-0809-186) and EN (EN14211) approval certificates.

## Supporting Information Available

Raw sensor data showing time-resolved Δλ_peak_ responses of Au nanodisks upon NO_2_ exposure and the Au–WO_3_ sensor upon NO_2_ exposure in dry synthetic air and humid air, concentrations of NO_2_ and NO measured using Serinus 40 during the field test, and response of the Au–WO_3_ sensor upon NO exposure (PDF)
