# Supplementary material for: Nanoplasmonic NO2 Sensor with a Sub-10 Parts per Billion Limit of Detection in Urban Air
Source: ACS Sens. 2022 Mar 31;7(4):1008–18. doi: 10.1021/acssensors.1c02463 (PMC9040054; doi:10.1021/acssensors.1c02463)
Supplement: Supplementary file 1 — se1c02463_si_001.pdf [file se1c02463_si_001.pdf]

Supporting Information

for

A Nanoplasmonic NO_2 Sensor with Sub-10 *parts per billion* Limit of Detection in Urban Air

Irem Tanyeli[1,2,*], Iwan Darmadi[1], Martin Sech[2], Christopher Tiburski[1], Joachim Fritzsche[1], Olof Andersson[2], Christoph Langhammer[1,*]

[1]*Department of Physics, Chalmers University of Technology, 412 96 Göteborg, Sweden.*

[2]*Insplorion AB, Arvid Wallgrens Backe 20, 413 46 Göteborg, Sweden.*

Corresponding authors: irem.tanyeli@insplorion.com; clangham@chalmers.se

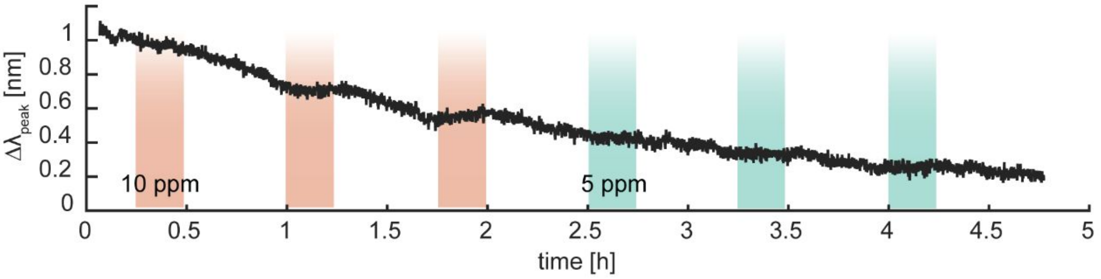

Figure S1*: Time-resolved $\Delta\lambda_{peak}$ response of Au nanodisks upon NO_2 exposures at concentrations of 10 ppm and 5 ppm in dry synthetic air. The sensor chip is heated to 250 °C.*

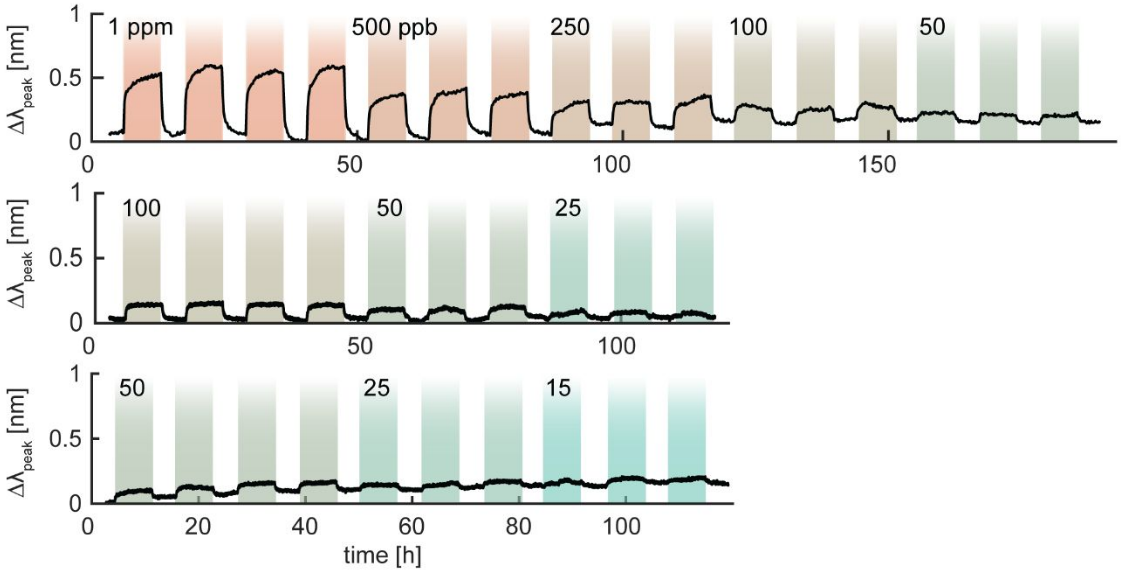

Figure S2. *Time-resolved $\Delta\lambda_{peak}$ response of the Au-WO_3 sensor upon pulsed exposure to different NO_2 concentrations in dry synthetic air. The sensor chip is heated to 250 °C. The shaded areas denote the pulses of NO_2 exposure with the specific concentrations indicated in the figure.*

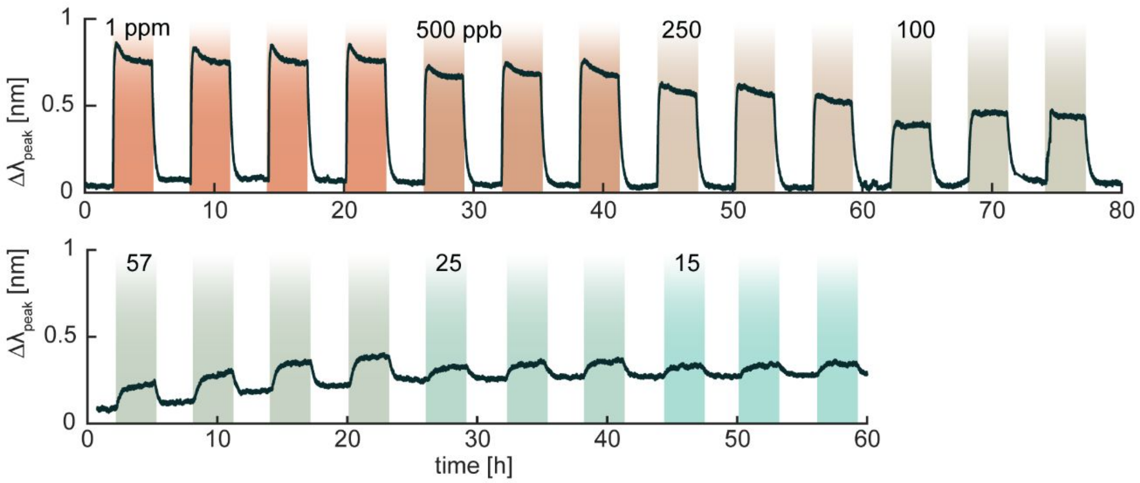

Figure S3. *Time-resolved $\Delta\lambda_{peak}$ response of the Au-WO_3 sensor upon pulsed exposure to different NO_2 concentrations in synthetic air permanently mixed with 1 ppm CO, 400 ppm CO_2 and 50% RH @ 30 °C. The sensor chip is heated to 150 °C. The shaded areas denote the pulses of NO_2 exposure with the specific concentrations indicated in the figure.*

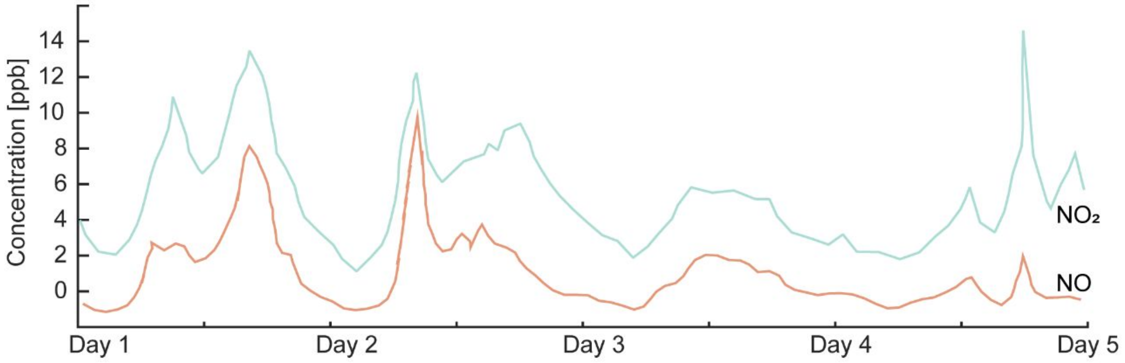

Figure S4. *The concentrations of NO_2 and NO measured by Serinus 40 during the five-day field test.*

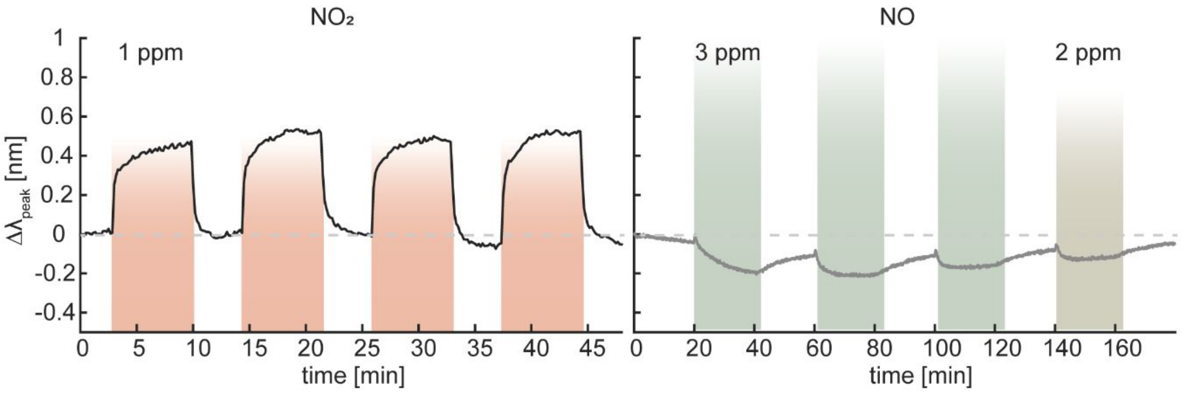

Figure S5. *Time-resolved $\Delta\lambda_{peak}$ response of a nanoplasmonic Au-WO_3 sensor upon exposure to NO_2 pulses at concentration of 1 ppm and NO pulses at concentrations of 3 ppm and 2 ppm in dry synthetic air. The sensor chip is heated to 250 °C.*
